# A Novel Method for 3D Lung Tumor Reconstruction Using Generative Models

**DOI:** 10.3390/diagnostics14222604

**Published:** 2024-11-20

**Authors:** Hamidreza Najafi, Kimia Savoji, Marzieh Mirzaeibonehkhater, Seyed Vahid Moravvej, Roohallah Alizadehsani, Siamak Pedrammehr

**Affiliations:** 1 Biomedical Engineering Department, School of Electrical Engineering, Iran University of Science and Technology, Tehran 16846-13114, Iran; ha_najafi@alumni.iust.ac.ir; 2 Biomedical Data Science and Informatics, School of Computing, Clemson University, Clemson, SC 29634, USA; ksavoji@g.clemson.edu; 3 Department of Electrical and Computer Engineering, Indiana University-Purdue University, Indianapolis, IN 46202, USA; marzieh89mirzaei@gmail.com; 4 Department of Electrical and Computer Engineering, Isfahan University of Technology, Isfahan 84156-83111, Iran; sa.moravvej@alumni.iut.ac.ir; 5 Institute for Intelligent Systems Research and Innovation (IISRI), Deakin University, Geelong, VIC 3216, Australia; r.alizadehsani@deakin.edu.au; 6 Faculty of Design, Tabriz Islamic Art University, Tabriz 51647-36931, Iran

**Keywords:** lung cancer, lung segmentation, tumor detection, 3D tumor reconstruction, generative adversarial network, imbalanced data

## Abstract

Background: Lung cancer remains a significant health concern, and the effectiveness of early detection significantly enhances patient survival rates. Identifying lung tumors with high precision is a challenge due to the complex nature of tumor structures and the surrounding lung tissues. Methods: To address these hurdles, this paper presents an innovative three-step approach that leverages Generative Adversarial Networks (GAN), Long Short-Term Memory (LSTM), and VGG16 algorithms for the accurate reconstruction of three-dimensional (3D) lung tumor images. The first challenge we address is the accurate segmentation of lung tissues from CT images, a task complicated by the overwhelming presence of non-lung pixels, which can lead to classifier imbalance. Our solution employs a GAN model trained with a reinforcement learning (RL)-based algorithm to mitigate this imbalance and enhance segmentation accuracy. The second challenge involves precisely detecting tumors within the segmented lung regions. We introduce a second GAN model with a novel loss function that significantly improves tumor detection accuracy. Following successful segmentation and tumor detection, the VGG16 algorithm is utilized for feature extraction, preparing the data for the final 3D reconstruction. These features are then processed through an LSTM network and converted into a format suitable for the reconstructive GAN. This GAN, equipped with dilated convolution layers in its discriminator, captures extensive contextual information, enabling the accurate reconstruction of the tumor’s 3D structure. Results: The effectiveness of our method is demonstrated through rigorous evaluation against established techniques using the LIDC-IDRI dataset and standard performance metrics, showcasing its superior performance and potential for enhancing early lung cancer detection. Conclusions:This study highlights the benefits of combining GANs, LSTM, and VGG16 into a unified framework. This approach significantly improves the accuracy of detecting and reconstructing lung tumors, promising to enhance diagnostic methods and patient results in lung cancer treatment.

## 1. Introduction

Lung cancer stands as a critical global health challenge, marking it as one of the deadliest cancers that claim the lives of millions worldwide annually, regardless of gender. For example, there were about 2.09 million new cases in 2018, leading to nearly 1.76 million deaths [1]. Detecting the disease early can substantially improve the chances of survival. However, despite the availability of sophisticated imaging tools such as CT scans, X-rays, and magnetic resonance imaging (MRI), inherent limitations hinder their effectiveness. CT scans offer detailed three-dimensional views of lung tissue; however, their high rates of false positives can lead to unnecessary biopsies and surgeries [2]. Although X-rays are commonly used for initial screenings, they lack the sensitivity to detect early-stage tumors and struggle to differentiate between benign and malignant nodules. MRIs deliver excellent contrast in soft tissues, yet their use in lung imaging is limited due to the lungs’ high air content and motion during breathing [3].

Integrating deep learning in medical imaging presents a promising frontier for overcoming these challenges. Deep learning models, trained on extensively annotated image datasets, have demonstrated remarkable capabilities in identifying subtle patterns that elude human radiologists and traditional machine learning models. These models can enhance the specificity and sensitivity of lung cancer detection, reduce the rate of false positives, and potentially decrease the need for invasive diagnostic procedures. In this article, we present a sophisticated deep-learning model tailored to construct the 3D structures of lung tumors accurately. This model leverages deep learning’s strengths to surpass the limitations of existing imaging technologies. The 3D lung tumor reconstruction process unfolds in three primary steps: lung segmentation, tumor identification and extraction, and reconstructing the tumor’s 3D shape using sequential images. This methodology sharpens the clarity of the tumor’s spatial configuration and provides crucial information that aids effective medical decision-making and treatment planning [4]. Through this research, we aim to showcase the application of deep learning in medical imaging and its potential to transform early detection and treatment.

A variety of methods have been developed for lung segmentation, which includes the use of hand-selected features and the careful tuning of experimental parameters [5]. However, these methods are often explicitly tailored for particular applications and datasets, limiting their generalizability, especially in medical imaging data like CT scans. This limitation is further exacerbated by the tendency to overlook certain features during the feature selection phase [6,7], as practitioners often skip detailed feature selection in favor of more intuitive interactive methods. Traditional machine learning approaches typically adopt a two-step process, beginning with feature selection and then applying a learning algorithm to build an automated system. Initially, experts choose or develop a method specifically for feature selection tailored to the task at hand [8]. In contrast, deep learning models integrate feature selection with the learning process, eliminating the need for separate feature selection algorithms [9,10,11,12]. Deep learning has been particularly effective in lung segmentation, focusing on classifying each pixel as either lung tissue or not. However, this pixel-wise classification approach is challenged by class imbalance, as the number of non-lung pixels significantly outnumbers lung pixels.

To address the class imbalance issue, strategies are employed at both the data handling and algorithmic levels [13,14]. Regarding data handling, techniques include downsampling the overrepresented classes, upsampling the underrepresented classes, or generating synthetic data to balance the dataset. At the algorithmic level, efforts focus on adjusting algorithms to recognize better and weigh the contributions of the less represented classes, which is essential for accurate predictions [15]. The primary problem with data-driven methods lies in their potential to reflect and amplify existing biases in the data, leading to a preference for more common outcomes and overlooking rare but essential cases [16]. From an algorithmic standpoint, the challenge is to adapt learning models to capture these vital, albeit infrequent, phenomena, thereby improving the accuracy and reliability of the model. The advent of RL has garnered considerable attention for its capability to address complex challenges, especially in scenarios marked by class imbalances [17]. RL enhances the efficacy of classification models by filtering out irrelevant details and emphasizing crucial features, demonstrating versatility across various domains. A notable strength of RL is its adaptive learning approach, guided by reward mechanisms, which is particularly effective in managing data imbalance issues [18]. By tailoring the reward system to prioritize accurately identifying underrepresented classes, RL strategies can focus more on these often-neglected segments. This method promotes a more equitable distribution of attention across different courses, significantly improving detection accuracy [19].

The 3D reconstruction from CT images has been thoroughly explored, significantly relying on statistical methodologies [20,21,22]. While these approaches offer considerable value, they encounter several notable limitations. One of the primary challenges is the dependency on extensive annotated datasets, which are notably difficult to obtain in medical imaging due to stringent privacy regulations and the necessity for annotations by medical experts. Additionally, these methods have a heightened risk of overfitting, compromising their ability to be applied effectively across varied medical datasets [23]. The assumptions made about data distribution in statistical methods may only sometimes align with the realities of complex medical environments, potentially leading to reconstruction inaccuracies [24]. The inherently high dimensionality of medical imaging data also presents computational hurdles, often requiring dimensionality reduction techniques that risk excluding crucial information. Moreover, statistical approaches may need help to accurately represent the intricate spatial relationships and textures, which is essential for precise 3D reconstructions in medical imaging [25].

GANs [26] present a transformative approach to overcoming the limitations inherent to traditional statistical methods for the 3D reconstruction of CT images, leveraging their distinctive architecture and learning paradigms. Central to the GAN framework is the symbiotic relationship between two deep neural networks: the generator and the discriminator [27]. The role of the generator is to create synthetic yet plausible data that mimic real CT images, whereas the discriminator evaluates these generated samples, striving to differentiate between the authentic and the synthetic [28]. This adversarial dynamic fosters a continuous learning cycle, driving both networks toward higher performance levels. The generator learns to produce increasingly realistic images, refining details to deceive the discriminator, while the discriminator enhances its ability to detect the subtleties that distinguish real images from fakes. This process generates high-quality, realistic 3D reconstructions that can be difficult to differentiate from actual CT scans.

This study presents an innovative triple-layered methodology for generating three-dimensional models of lung cancer from CT scans. The process begins with segmenting the CT images using a GAN incorporating the U-net architecture, enabling precise pixel-level analysis. To tackle the class imbalance issue, we utilize an RL algorithm that prefers less represented classes, enhancing the sensitivity of the model to rarer but crucial features. The discriminator network, equipped with dilated convolution layers, improves the quality of the generator’s output by effectively differentiating between authentic and synthesized images and emphasizing key features. In the subsequent phase, we employ another GAN setup with the Mask R-CNN framework, which accurately identifies and localizes affected regions within the segmented images, thereby facilitating precise tumor detection and classification. To further refine the accuracy of these identified areas, an error correction component is integrated into the generator’s loss function, and the discriminator continues to use dilated convolution layers for high-quality output validation. The final stage of the process involves 3D modeling, where LSTM networks combined with GAN technology play a pivotal role. Here, features extracted from two-dimensional pulmonary scans by a trained VGG16 network are further processed by an LSTM network, which employs an attention mechanism to highlight critical tumor-related features. The generator then creates a three-dimensional representation based on the output of the LSTM. At the same time, the discriminator evaluates the fidelity of these 3D models against real images, ensuring the reconstructed models closely mirror actual lung structures. The efficacy of our method is validated through comprehensive testing, benchmarked against well-established approaches using the LIDC-IDRI dataset [29] and conventional performance metrics. This comparison highlights our method’s superior capabilities and promising potential to improve the early detection of lung cancer.

The proposed model in this study offers several significant contributions to the field of medical imaging, specifically in the 3D reconstruction of lung cancer from CT scans:

- Addressing class imbalance with RL: Traditional methods for lung segmentation often need help with class imbalance, where certain lung conditions are significantly underrepresented in training datasets. Our approach integrates RL to target this issue specifically. Using RL, the model dynamically adjusts its focus towards these less-represented classes during training, enhancing its sensitivity and specificity in these areas. This targeted focus mitigates the class imbalance problem and improves the model’s generalization capabilities across diverse patient datasets.
- Innovative reward mechanism in RL for enhanced lung segmentation: We have developed an innovative reward mechanism within our RL strategy tailored for lung segmentation. This mechanism provides a higher reward for correctly identifying typically underrepresented classes. This approach ensures that the model pays greater attention to these infrequent classes, promoting a more equitable and balanced segmentation process.
- Utilization of dilated convolution in discriminator network: Our model incorporates a discriminator network that employs dilated convolution layers. This architectural choice allows the network to expand its receptive field without losing resolution, enabling it to better differentiate between real and synthesized images. The ability to focus on more extensive and critical features significantly improves the reconstructed images’ visual clarity and detail resolution.
- Error correction in generator’s loss function for tumor detection: We have integrated an error correction component into the loss function of our generator during the tumor detection phase. This component is specifically designed to fine-tune the detection accuracy and minimize potential errors in the output. By implementing this correction process, the model enhances its reliability and ensures high precision in tumor detection.

The remainder of the article is organized as follows: Section 2 discusses the related work, Section 3 details the proposed methodology, Section 4 describes the experiments conducted, and Section 5 provides the conclusion and outlines future research directions.

## 2. Related Work

The literature review is divided into three sections: lung segmentation, tumor detection, and 3D tumor reconstruction.

### 2.1. Lung Segmentation

Hasan et al. [30] delve into deep learning applications for diagnosing diseases such as COVID-19 and tuberculosis by analyzing pulmonary X-rays, explicitly employing the deeplabv3plus CNN-based semantic segmentation model that utilizes “Atrous Convolution” to preserve feature resolution in deeper layers of the network. Swaminathan et al. [31] tackle the pressing issue of diagnosing lung cancer at advanced stages by employing deep learning to improve early detection rates, particularly in high-risk individuals. Their methodology involves initial preprocessing of CT images with the Wiener filter, followed by segmentation using a GAN trained with the Salp Shuffled Shepherd Optimization Algorithm (SSSOA), and subsequent classification via the VGG16 CNN model. Gite et al. [32] present an innovative approach to lung segmentation in X-ray images using U-Net++, aiming to boost tuberculosis detection rates. This novel method surpasses traditional techniques and mitigates data leakage problems, significantly enhancing diagnostic precision. Irawan et al. [33] unveil a modified U-Net architecture tailored for semantic lung segmentation from chest X-rays, integrating multiple dropouts in deconvolutional layers to avoid overfitting, thereby ensuring high accuracy and model generalization with a streamlined framework. Rehman et al. [34] validate the U-Net framework’s capability in segmenting lung areas from X-ray images, attaining a notable average Intersection over Union (IoU) score of 92.82. Shaoyong Guo et al. [35] introduce a sophisticated automated segmentation method that merges radionics with manually selected and algorithmically derived features, achieving a Dice similarity index of 89.42% on the ILD database MedGIFT. Chen Zhou et al. [36] develop an advanced automatic segmentation system that incorporates a 3D V-Net and a Spatial Transform Network (STN) to accurately segment lung tissue in CT scans, facilitating the diagnosis of COVID-19 by examining textures and other critical features. Mizuho Nishio et al. [37] utilize a U-Net framework optimized through Bayesian methods on datasets from Japan and Montgomery, securing high Dice Similarity Coefficients (DSC) of 0.976 and 0.973, respectively. Ferreira et al. [38] propose a unique U-Net variant designed for the automated detection of COVID-19 infections in CT scans, validated on actual patient data from Pedro Ernesto University Hospital in Rio de Janeiro. Feidao Cao [39] enhances the conventional U-Net structure by incorporating a Variational Auto-Encoder (VAE) in each encoder–decoder layer, markedly improving the network’s feature extraction efficiency. This advanced network is evaluated using NIH and JRST datasets, demonstrating exceptional accuracy and F1 scores of 0.9701 and 0.9334 for the former and 0.9750 and 0.9578 for the latter, respectively.

Many current lung segmentation techniques involve classifying each pixel as either lung tissue or not, which presents a challenge due to class imbalance; the overwhelming majority of pixels represent non-lung tissue. Acknowledging this issue, our paper introduces an RL-based innovative approach to lung segmentation that circumvents the problem of unbalanced classification.

### 2.2. Tumor Detection

Vijh et al. [40] introduced a hybrid bio-inspired algorithm named WOA_APSO, which merges the whale optimization algorithm (WOA) with adaptive particle swarm optimization (APSO) to refine feature selection in a CNN for lung CT image classification, thereby improving tumor detection accuracy. Rathod et al. [41] developed DLCTLungDetectNet, a deep learning framework that employs a CNN with FusionNet, integrating features from ResNet50 and InceptionV3, to enhance early lung cancer detection in CT scans. Their model outperforms traditional architectures like VGG16 and Inception v3, setting a new standard in automated lung tumor detection. Venkatesh et al. [42] proposed a novel method for early lung cancer detection in CT images using patch processing and deep learning to classify tumors efficiently, aiming for accurate detection with less computational time and improved performance metrics. Sundarrajan et al. [43] introduced an advanced lung tumor detection approach leveraging cloud-based IoT for data collection and employing an optimized fuzzy C-means neural network (OFCMNN) for segmentation, followed by classification with a kernel multilayer deep transfer convolutional learning (KM-DTCL) ensemble to provide superior diagnostic performance. Srinivasulu et al. [44] developed a deep learning model, ECNN-ERNN with autoencoders, for early detection of lung malignancies in CT images, utilizing a modified framework to achieve high accuracy and insightful feature extraction. Manickavasagam et al. [45] proposed CNN-5CL. This novel deep learning methodology uses an 11-layer CNN, including five convolutional layers, for precise pulmonary nodule classification in CT images, benchmarked with LIDC/IDRI datasets. Agarwal et al. [46] introduced a method that combines a CNN with the AlexNet Network Model for early lung tumor classification, distinguishing malignant from benign tumors with greater accuracy and addressing the challenge of premature lung cancer diagnosis. Li and Fan [47] unveiled a 3D CNN-based pulmonary nodule detection model featuring an encoder–decoder architecture, a dynamically scaled cross-entropy method for reducing false positives, and a squeeze-and-excitation structure to capitalize on channel dependencies.

### 2.3. Three-Dimensional Tumor Reconstruction

Recent advancements in the medical field have led to the development of 3D models, significantly enhancing patient treatment strategies [48,49]. A prime example of this application is in liver resection surgeries, where 3D models are crucial in providing surgeons with a detailed understanding of liver anatomy [50]. Furthermore, the creation of 3D brain models using magnetic resonance imaging (MRI) has been investigated [51]. The predominant emphasis in current research revolves around the 3D reconstruction of lung tumors [52,53]. Afshar et al. [21] proposed a method for segmenting lung cancer tumors and reconstructing 3D CT images. Their approach used snake optimization for lung segmentation, Gustafson and Kessel (GK) clustering [54] for tumor detection, and a statistical technique for 3D reconstruction. Hong et al. [22], and Rezaei et al. [55] employed a similar method for lung and tumor detection but with including a GAN-based approach for reconstructing 3D images. However, the accuracy of snake optimization heavily relies on the initial positioning of the contour [56]. If the initial contour placement is correct or deviates from the actual lung boundaries, it can lead to accurate segmentation results. Snake optimization may face challenges when segmenting complex lung structures, such as irregular shapes, nodules, or lesions, as these variations can make it difficult for the contour to adapt and accurately capture their boundaries. Similarly, GK clustering, like other clustering algorithms, is sensitive to the initial selection of cluster centroids. Choosing appropriate initial centroids is crucial to obtain accurate tumor detection. Poorly chosen centroids can cause suboptimal clustering outcomes. GK clustering may also struggle to handle cases where tumors overlap or exhibit heterogeneous characteristics, making it challenging to differentiate between different tumor regions and assign data points accurately to their respective clusters [52].

## 3. The Proposed Model

**Figure 1:**
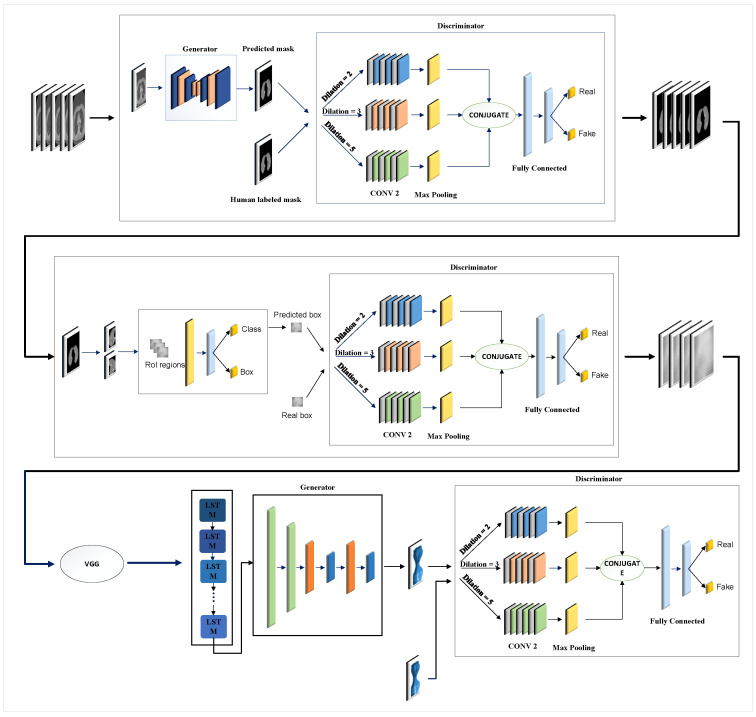
Overview of the proposed model: In step 1, two lungs are segmented in CT images using the GAN-based model. In step 2, the tumor is detected using the second GAN-based model. After the features are extracted by VGG16, a 3D model of the tumor is reconstructed using the third GAN in step 3.

Figure 1 illustrates the overall structure of the proposed model, which encompasses three key steps: lung segmentation, tumor detection, and 3D tumor reconstruction. In the subsequent sections, we will discuss each step, elaborating on their functionalities and processes.

### 3.1. Lung Segmentation

The primary objective of segmentation in medical imaging is to produce precise lung outlines that closely mirror the reference or original outline. These outlines are pivotal for many medical uses and further examinations by accurately defining the edges of the lungs. To achieve this, we utilize a duo of neural networks: a generator (G) and a discriminator (D). G is crucial in crafting lung outlines, taking black-and-white CT scans as input. Through its complex structure, G is adept at identifying significant features and patterns within the images, creating outlines that suggest the areas of the lungs. These outlines endeavor to accurately capture the complex shapes and perimeters of the lungs, which is crucial for detailed segmentation. Conversely, D collaborates with G to boost the precision and quality of these outlines. Acting as a rigorous assessor, D examines how closely the created outlines match the reference or original outlines. Utilizing sophisticated methods like the Earth Mover’s (EM) distance, D measures the variance between the predicted and the reference outlines, enhancing G’s output. This motivates G to produce outlines that align with the original, improving the segmentation outcomes. During the training phase, G is fed a lung CT scan image, referred to as $I_{i}$, and generates a corresponding outline $M_{i}$ that highlights the areas of the lungs. D then scrutinizes the created outline $M_{i}$ for its fidelity and quality, offering critical feedback that aids G’s learning and advancement. This dynamic interaction between G and D, coupled with reference outlines, aids in continuously enhancing the lung outlines generated through the training. In the forthcoming sections, we will explore the intricacies of G and D’s designs, elucidating their structure and functionalities. We will also discuss the loss function utilized during training, which is significant in steering the networks toward yielding more precise and dependable lung segmentation.

#### 3.1.1. Generator Architecture

**Figure 2:**
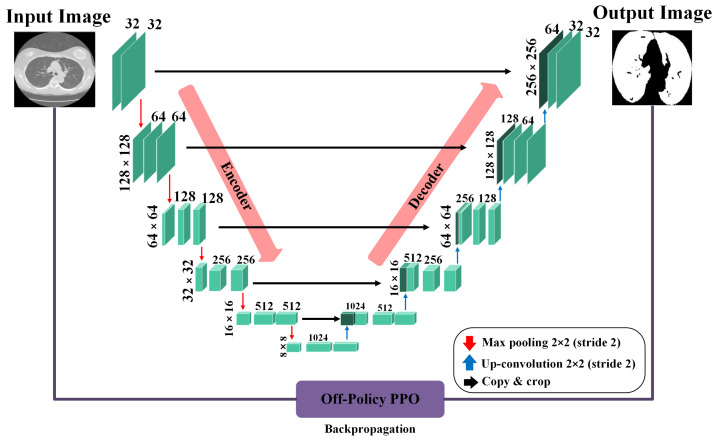
Architecture of the U-Ne-based generator network used for lung segmentation, illustrating the flow from input CT scan through the encoder and decoder stages to the final mask output.

In our context, segmentation is treated as a task where each pixel in an image is classified as either part of the lung area or not. To perform this task, we rely on the precision of a U-Net-based generator network, as depicted in Figure 2. When processing a CT scan labeled $I_{i}$, the generator network (G) meticulously evaluates each pixel to determine its association with the lung region, subsequently generating a mask $M_{i}$ that accurately reflects this classification. The generator network has two principal components: the encoder and the decoder. The encoder, in its complex task, begins by taking the input image $I_{i}$ and, through a series of convolution layers, extracts features at multiple scales. These layers enable the encoder to delve into the image, capturing a broad range of details and complex information from the image. Following the feature extraction, the decoder, playing a pivotal role, uses these multi-level features to construct the masks. By leveraging these features, the decoder can accurately reconstruct the intricate structures and contours of the lung area, resulting in detailed and precise masks. Both the encoder and decoder sections include convolution layers, which are crucial in neural network designs for identifying local patterns and spatial correlations, and are key elements for accurate segmentation.

The generator network is designed as a binary pixel classifier, identifying each pixel as either part of the lung area (labeled as one) or not (labeled as zero). A significant challenge arises due to the overwhelming number of pixels labeled as zero, which is not associated with the lung area and leads to an imbalance. This imbalance causes the classifier to lean towards predicting pixels as non-lung areas, which can drastically reduce the model’s ability to delineate the lung region accurately. To counteract this issue, the training strategy for the generator incorporates an RL algorithm. RL is based on reward and punishment mechanisms, making it an ideal solution for addressing the problem of imbalanced classification. Within this framework, the RL algorithm is customized to assign much higher rewards for accurately identifying pixels within the minority class (the lung region) than those for correctly classifying pixels in the majority class (non-lung area). Conversely, the penalties for incorrectly classifying lung region pixels are more stringent than errors made within the majority class. Despite their scarcity, this strategy shifts the model’s focus towards more accurately recognizing and classifying pixels within the lung region. By amplifying the consequences for the minority class, the RL algorithm motivates the generator network to concentrate more on lung areas, thus mitigating the bias towards the more prevalent non-lung pixels. This adjustment not only equalizes the classifier’s attentiveness to both classes but also significantly boosts the model’s precision and effectiveness in segmenting lung regions from CT scans.

The loss function for the generator is defined as follows [57]:

(1) $$\theta_{g}^{\prime }=\theta_{g}+\alpha \sum_{t=0}^{T}\nabla_{\theta_{g}}\log\pi_{\theta_{g}}\left(a_{t}|s_{t}\right)\bar{R_{t}}$$

where $\theta_{g}$ and $\theta_{g}^{\prime }$ represent the parameters of the generator before and after training, respectively. α is the learning rate, while *T* is a terminal time step. $\nabla_{\theta_{g}}$ signifies the gradient with respect to the generator parameters. $\log\pi_{\theta_{g}}\left(a_{t}|s_{t}\right)$ is the logarithm of the policy defined by the generator parameters, which gives the probability of taking action $a_{t}$ in state $s_{t}$. $\bar{R_{t}}$ represents the average reward received at time *t*, calculated across all pixels, where $R_{t}$ is defined as follows [58]:

(2) $$R_{t}\left(s_{t},a_{t},y_{t}\right)=\left\{\begin{array}{cc} +1, & \mathrm{if}\;a_{t}=y_{t}\;\mathrm{and}\;s_{t}\in D_{L}\\ -1, & \mathrm{if}\;a_{t}\neq y_{t}\;\mathrm{and}\;s_{t}\in D_{L}\\ +\lambda , & \mathrm{if}\;a_{t}=y_{t}\;\mathrm{and}\;s_{t}\in D_{N}\\ -\lambda , & \mathrm{if}\;a_{t}\neq y_{t}\;\mathrm{and}\;s_{t}\in D_{N} \end{array}\right.$$

where $D_{L}$ and $D_{N}$ represent the minority (lung) and majority (no lung) classes, respectively. Correctly/incorrectly classifying a sample from the majority class yields a reward of ±λ, where 0<λ<1. The pseudocode for our proposed method is outlined in Algorithm 1.
**Algorithm 1** Pseudocode of RL-based model for lung segmentation.  1:Initialize generator parameters $\theta_{g}$ and learning rate α.  2:Load training dataset consisting of CT scan images and corresponding ground truth masks.  3:**while** not converged **do**  4:     **for** each image $I_{i}$ in the dataset **do**  5:         Process $I_{i}$ through generator to produce mask $M_{i}$.  6:         Compute rewards $R_{t}$ for each pixel based on its classification:
$$R_{t}\left(s_{t},a_{t},y_{t}\right)=\left\{\begin{array}{cc} +1, & \mathrm{if}\;a_{t}=y_{t}\;\mathrm{and}\;s_{t}\in D_{L}\\ -1, & \mathrm{if}\;a_{t}\neq y_{t}\;\mathrm{and}\;s_{t}\in D_{L}\\ +\lambda , & \mathrm{if}\;a_{t}=y_{t}\;\mathrm{and}\;s_{t}\in D_{N}\\ -\lambda , & \mathrm{if}\;a_{t}\neq y_{t}\;\mathrm{and}\;s_{t}\in D_{N} \end{array}\right.$$  7:         Calculate gradients of the policy $\log\pi_{\theta_{g}}\left(a_{t}|s_{t}\right)$.  8:         Update $\theta_{g}$ using policy gradient update rule:
$\theta_{g}^{\prime }=\theta_{g}+\alpha \sum_{t=0}^{T}\nabla_{\theta_{g}}\log\pi_{\theta_{g}}\left(a_{t}|s_{t}\right)\bar{R_{t}}$  9:     **end for**10:**end while**

#### 3.1.2. Discriminator Architecture

The discriminator network plays a pivotal role in differentiating between the masks created by the generator and the genuine masks. The Earth Mover’s (EM) distance is used as an effective and smooth measure to gauge the difference between the true and generated mask distributions. The discriminator approximates the EM distance by evaluating the gap in expected values between samples from the model’s distribution $p_{z}$ and the actual distribution $p_{real}$. This is conducted by analyzing the differences in the outputs from $D_{x∼p_{z}}\left(G\left(x\right)\right)$ and $D_{x∼p_{real}}\left(x\right)$. Unlike the conventional discriminator in a Generative Adversarial Network (GAN), which is typically tasked with classification, our discriminator assumes a regression function. Its goal is to approximate the EM distance function precisely. In tasks like image generation with GANs, the aim is often to create images that closely mimic real ones. However, in lung segmentation, the challenge is different. The mask generated by the generator varies in intensity across a continuum from 0 to 1, in contrast to the mask, which is strictly binary, consisting entirely of 0 s and 1s. This difference could cause the discriminator to focus too narrowly on distinguishing between real and generated masks, possibly missing other important aspects. To avoid this, we do not directly input the generator’s output or the real masks into the discriminator. Instead, we use a modified approach where the discriminator’s input includes lung slice images. These images are altered versions of the original CT scans, modified using segmented and actual masks. This technique allows the discriminator to evaluate the relationship between the generated masks and the original lung images, thus improving its ability to distinguish between real and generated masks accurately. Additionally, a segmentation mask is used as extra information to help maintain the nodule regions in the original image. This different mask selectively retains nodule areas while setting the rest to 0. With this additional detail, the discriminator can better identify important features, enhancing its ability to differentiate effectively. The loss function for the discriminator is articulated using the subsequent equation:

(3) $$E_{x∼p_{z}}\left[D\left(G\left(x\right)oI_{i}\right)\right]-E_{x∼p_{real}}\left[D\left(x\right)\right]$$

Here, the symbol *o* represents an operator that performs pixel-wise multiplication.

### 3.2. Tumor Detection

Figure 1 illustrates the critical component of our research, the generator network, which employs a Mask R-CNN framework. Moving away from the traditional approach of GANs that typically use random noise to create synthetic outputs, our model adopts a more strategic method. Specifically, it utilizes detailed lung data obtained from an earlier phase as its input rather than relying on random noise. This strategic choice of input allows our generator to capitalize on the comprehensive information within the lung data to craft precise and relevant outputs. The generator’s main objective is to deliver outputs that include detailed classification and location data. By processing the lung data, the generator can discern different regions within the lungs, assign appropriate class labels to these regions, and accurately determine their locations with bounding box coordinates. To steer the training of the generator and enhance its output accuracy, we introduce a specialized loss function, noted as Equation (4). This loss function evaluates the difference between the generator’s outputs and the actual verified data. By employing this loss function, our framework methodically improves the generator’s proficiency in producing precise classification and location details for the lung data it processes.

(4) $$L_{G}=L_{cls}+L_{box}+L_{adv}^{G_{b}}$$

In this context, $L_{cls}$ and $L_{box}$ are the loss components used within the Mask R-CNN framework, targeting the accuracy of class predictions and the precision of bounding box placements, respectively. Equation (5) introduces the adversarial loss component $L_{adv}^{G_{b}}$, which is instrumental in refining the performance of the generator.

(5) $$L_{adv}^{G_{b}}=\frac{1}{N}\Sigma_{i=1}^{N}-\log D_{b}\left(G_{b}\left(RoI_{i}\right)\right)$$

Here, *N* is the mini-batch size, and $G_{b}\left(RoI_{i}\right)$ represents the *i*-th bounding box prediction. The discriminator is critical in assessing the quality of bounding boxes proposed by the generator. However, evaluating the boxes based solely on their coordinates may not provide a thorough assessment. To overcome this, a method is suggested where feature maps for both authentic and generated boxes are presented together, allowing for a more nuanced evaluation of the bounding box quality. The discriminator’s loss function, as laid out in Equation (6), is crucial for its training. It integrates three Convolutional Neural Networks (CNNs) that collaborate to enhance the discriminator’s ability to differentiate between authentic and fabricated bounding boxes, thereby aiding overall training efficacy.

(6) $$L_{D}=\frac{1}{N}\Sigma_{i=1}^{N}-\left[\log\left(D_{b}\left(bb_{i}^{g_{t}}\right)\right)+\log D_{b}\left(1-D_{b}\left(G_{b}\left(RoI_{i}\right)\right)\right)\right]$$

In this formulation, $G_{b}\left(RoI_{i}\right)$ denotes the actual parameters for the *i*-th bounding box, while $D_{b}$ indicates the likelihood that the image is authentic. The adversarial loss $L_{adv}^{G_{b}}$ incentivizes $G_{b}$ to create bounding boxes convincing enough to mislead $D_{b}$.

### 3.3. The 3D Reconstruction Method

As Figure 1 shows, segmenting tumors results in *N* series of 2D slices. These slices undergo feature extraction using the VGG-16 network. The feature vectors from VGG-16 simplify the data by reducing their dimensionality, making them suitable for sequential analysis by the LSTM. This method enables the LSTM to effectively analyze the sequence of slices, which is crucial for accurate 3D tumor reconstruction while avoiding issues like overfitting and the high computational demands associated with processing large image dimensions directly. The outputs from VGG-16 feed into the LSTM, whose results are then employed by a GAN framework for further processing. The GAN framework is designed to create 3D images from the sequences of 2D slices. In this setup, the GAN discriminator differentiates between the images generated by its generator and real images.

## 4. Empirical Evaluation

In the upcoming section, we describe the dataset, detailing its features and scope as used in our study. This is followed by an explanation of the metrics, where we specify the criteria and measurements employed to assess our models’ performance. The section concludes with the presentation of results, highlighting the key findings from our analysis and model evaluations and discussing their significance in the context of our research objectives.

### 4.1. Dataset

The Lung Image Database Consortium image collection (LIDC-IDRI) [59] was developed through a collaboration between the Foundation for the National Institutes of Health (FNIH) and the Food and Drug Administration (FDA). This valuable dataset includes 1018 CT scans from 1010 individuals, each paired with an XML file. The XML file holds detailed annotations made by four experienced radiologists to meticulously identify and catalog every nodule visible in the scans. The annotation process is divided into two main phases to ensure thoroughness. In the first phase, known as the blinded-read phase, each radiologist independently reviews the scans to pinpoint lesions, marking those considered as “nodule < 3 mm” and “non-nodule ≥ 3 mm”. Following this, in the unblinded-read phase, they revisit their initial findings and consider their peers’ anonymous annotations. Within this dataset, a total of 7371 lesions were marked as nodules by at least one reviewing radiologist. Of these, 2669 lesions were unanimously classified as “nodule 3 mm” by all four radiologists, with 928 lesions consistently identified by every radiologist involved. These 2669 lesions were then subjected to detailed assessments, including intellectual nodule evaluations and the provision of precise nodule outlines. It is important to note that the original LIDC-IDRI dataset does not include 3D images. To bridge this gap, a meticulous manual process was employed using Rhinoceros 3D software, enabling the creation of 3D representations for these images. Rhinoceros 3D, version 8.0, developed by Robert McNeel, is sourced from Seattle, Washington, United States of America [60].

In addition to the details about the LIDC-IDRI dataset, it is important to outline the preprocessing steps to prepare the data for our analysis. Initially, DICOM images were converted into a more accessible format using the PyDicom library, a Python-based tool that facilitates seamless integration and manipulation within our analysis framework. Each CT scan underwent normalization to standardize pixel intensity values, enhancing data consistency for our machine-learning models.

To enhance the quality and reliability of the data used in training, we applied data augmentation techniques, including rotation, zoom, and horizontal flipping. These methods increased our model’s robustness against new, unseen data variations. Subsequently, each augmented image was resized to uniform dimensions, streamlining processing and ensuring consistency in the input to convolutional neural networks. Additionally, the annotations from the radiologists were precisely converted into mask images that accurately represent the locations of the nodules in the CT scans, providing a reliable ground truth for supervised learning. These crucial preparatory steps ensured that the dataset was optimally prepared for achieving high-performance results in our study.

### 4.2. Metrics

We employ Intersection over Union (IoU) and Hausdorff Distance (HD) to assess the novel lung segmentation and tumor detection models as our primary evaluation metrics. IoU is particularly apt for segmentation tasks as it quantitatively measures the overlap between the predicted segmentation and the ground truth. This metric highly indicates the accuracy of the model in delineating the boundaries of lung regions and tumors, providing a clear picture of how well the model can identify and segregate the target areas from the surrounding tissues. HD, on the other hand, is utilized to gauge the spatial discrepancy between the predicted and actual boundaries of the tumors. This metric is especially suitable for evaluating the precision of tumor detection models, as it reflects the maximum distance of any point on the predicted boundary from the nearest point on the actual boundary. The smaller the HD value, the closer the prediction of the model is to the true shape of the tumor, indicating a higher level of accuracy in capturing the tumor’s geometric nuances [52].

For the proposed 3D reconstruction model, we continue to use HD along with Euclidean Distance (ED). HD remains a crucial metric due to its effectiveness in measuring the geometric fidelity of the reconstructed tumor shapes compared to real tumors. It ensures that the reconstructed models closely mirror the tumor shapes, maintaining the integrity of critical geometric features. ED complements HD by offering a scalar representation of the straight-line distance between corresponding points on the predicted and true surfaces. This metric is particularly relevant for 3D reconstructions as it provides insight into the average positional accuracy across the entire reconstructed tumor volume. By assessing both the maximum and average deviations (through HD and ED, respectively), we can understand the performance of the model in replicating the complex 3D structures of tumors, ensuring the reconstructions are not only precise in shape but also accurate in spatial positioning.

IoU, HD, and ED metrics are defined as follows:

(7) $$IoU=\frac{\left|A\cap B\right|}{\left|A\cup B\right|}$$

where *A* denotes the set of pixels in the ground truth mask, and *B* represents the pixels in the predicted mask.

(8) HD(A,B)=max(h(A,B),h(B,A))

where

(9) $$h\left(A,B\right)=\max_{a\in A}\min_{b\in B}∥a-b∥$$

In this context, *A* and *B* are the sets of non-zero pixels in the ground truth and predicted segmentation, respectively, and ∥a−b∥ denotes the Euclidean distance between points *a* and *b*.

(10) $$ED=\sqrt{{\left(x_{2}-x_{1}\right)}^{2}+{\left(y_{2}-y_{1}\right)}^{2}+{\left(z_{2}-z_{1}\right)}^{2}}$$

Here, $\left(x_{1},y_{1},z_{1}\right)$ and $\left(x_{2},y_{2},z_{2}\right)$ represent the coordinates of two points in the 3D space.

### 4.3. Main Results

To fine-tune parameters for lung delineation, lesion identification, and three-dimensional reconstruction methods, we employed stratified partitioning validation. This highly effective method ensures the resilience and transferability of our approach. This process involved dividing the dataset into equal parts and iterating the training cycle for each division, using one as the test set and the others for training. We conducted systematic experiments to isolate the impact of individual parameters by varying one at a time while keeping others constant. This approach allowed us to identify which parameters most significantly influenced the effectiveness of our methods.

Stratified partitioning validation ensured that each dataset split faithfully, and reflected the entire dataset’s overall variety and class proportions. This meticulous approach to dataset handling enhanced the model’s robustness by training it across a wide range of cases and minimized the potential for overfitting. By exposing the model to diverse yet statistically similar data subsets, we could consistently evaluate its performance across various scenarios. This method of repeated cycles of training and validation allowed us to incrementally refine our model, boosting its accuracy and dependability while preserving its generalization capabilities. Additionally, the stratified approach was crucial for detecting anomalies or biases in the dataset, enabling timely adjustments before the model’s finalization. Thus, this comprehensive validation strategy was pivotal in ensuring that our model delivered a strong performance on training and unseen data, effectively reducing the likelihood of overfitting.

The results of these parameter tuning exercises are summarized in Table 1. This table provides a detailed overview of the optimized parameters, outlining the ranges and specific values that achieved the highest efficacy during the testing phase.

During the training of our GANs and LSTM network, addressing the challenge of local minima was crucial for robust performance. We implemented several strategies to mitigate this risk. Initially, we initialized the GAN models with weights from a truncated normal distribution to stabilize the early training phase and prevent skewed feature influence. We used the Adam optimizer, known for its ability to handle the decay of past gradients and navigate complex parameter spaces effectively, thus avoiding local minima. For the LSTM network, dropout layers were strategically included to prevent overfitting. These approaches, alongside continuous validation checks, ensured our models avoided local minima and maintained excellent generalization across new datasets.

#### 4.3.1. Lung Segmentation

Our GAN-based model performs exceptionally precisely segmenting images, especially in accurately delineating lung regions against the surrounding anatomy. To evaluate its effectiveness, we conducted a detailed comparison with six cutting-edge models: Deeplabv3plus [30], U-Net [33], U-Net++ [32], LDDNet [61], LGAN [62], and 3D Res U-Net [63], with the comparative results encapsulated in Table 2. Within the range of models compared, Deeplabv3plus takes the lead with an IoU score of 0.798, indicating the highest degree of overlap between its predicted segmentation and the actual ground truth. Close contenders, U-Net and U-Net++, exhibit strong segmentation capabilities with IoU scores of 0.762 and 0.750, respectively. In contrast, LDDNet and LGAN show less precision in their segmentations, reflected in lower IoU scores of 0.662 and 0.632. However, when considering the Hausdorff Distance, LGAN and LDDNet outperform others with the lowest scores, suggesting that their segmentations are closer to the ground truth at their furthest point. Interestingly, 3D Res U-Net, despite its lower IoU, presents the highest HD score of 2.835, hinting at potential irregularities in its segmentation boundaries. Our proposed GAN-based model, however, marks a significant advancement with an IoU of 0.972, indicating a 21.8% improvement over the best-performing comparison model, and an HD score of 0.925, the lowest of the group, which translates to a 67.4% improvement from the highest HD score of the compared models. The comparison becomes even more striking when our model is evaluated against its variant without RL. The incorporation of RL has led to a notable IoU increase from 0.726 to 0.972, a leap of 33.9%, signifying a superior match with the ground truth. Similarly, the enhancement in HD from 1.205 to 0.925, a reduction of 23.2%, underscores the heightened accuracy of boundary detection with RL integration.

To ascertain the statistical significance of our proposed model’s superiority over existing models, we conducted paired *t*-tests on IoU and HD metrics. The tests yielded *p*-values such as 0.002 for IoU when comparing our model to Deeplabv3plus and 0.005 for HD when comparing to LGAN, indicating significant improvements. These results confirm the enhancements are significant and statistically robust, supporting the model’s superior performance. Additionally, the 95% confidence intervals, like those ranging from 0.15 to 0.21 for IoU differences with Deeplabv3plus, affirm these findings. This statistical evidence strongly validates the proposed model’s utility in clinical settings for more accurate and reliable lung segmentation.

**Figure 3:**
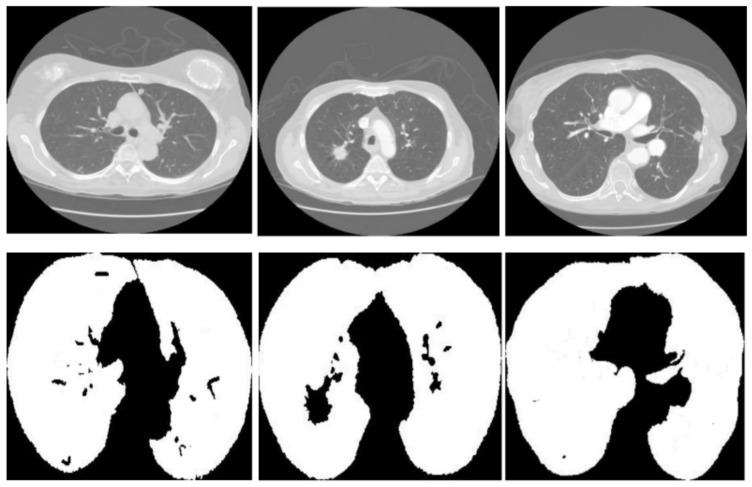
Comparative visualization of original CT scans and segmented lung regions by the proposed model.

Figure 3 displays three sets of images produced by the proposed lung segmentation model. The top row illustrates the original CT scans from patients, depicting cross-sectional images of the thoracic cavity. These scans provide a clear view of the lung structures, bones, and surrounding tissues, with radiodensity variations allowing these components’ differentiation. In the corresponding images of the bottom row, the results of the segmentation model are evident. Here, the lung regions have been delineated and filled in, starkly contrasting with the non-lung areas, which appear as black space. The segmentation model seems to have successfully extracted the lung regions from the CT images with high accuracy. In the first column, the model accurately identifies the lung boundaries and the separation between the left and right lungs. The second column shows a similarly precise segmentation, with the model correctly excluding the heart and the spine, which are centrally located in the thoracic cavity. In the third column, despite the more complex lung structures due to either a pathological condition or a scanning artifact, the model has managed to delineate the lung region, preserving the intricate details and the shape of the lungs.

**Figure 4:**
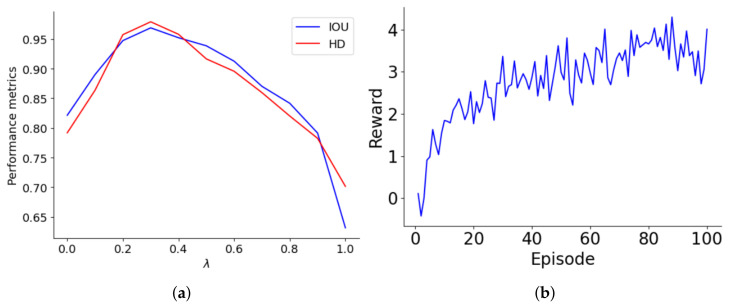
(**a**) Optimization of the λ parameter for lung segmentation model performance, (**b**) learning trajectory of the reward optimization of the agent on 100 episodes.

The reward structure of the lung segmentation model differentiates between accurate and inaccurate classifications, assigning values of ±1 and ±λ for the majority and minority classes, respectively. The optimal λ is tied to the class ratio and decreases as the majority-to-minority ratio increases. Performance was systematically evaluated over λ values ranging from 0 to 1 in 0.1 increments, with this assessment graphically represented in Figure 4a. As λ increases to about 0.4, both IoU and HD improve, indicating better accuracy for minority class predictions. However, beyond a λ of 0.4, performance declines, suggesting that too high a reward for the minority class may cause bias, leading to model overfitting or underfitting. The performance drop-off becomes significant as λ approaches 1, where the model heavily penalizes misclassifications of minority class samples, adversely affecting its generalization and accuracy.

Figure 4b illustrates the progression of the agent’s rewards over a series of episodes, highlighting its learning trajectory. Initially, the agent starts with a basic understanding, earning minimal rewards. As the episodes progress, there is a notable increase in rewards, especially between episodes 0 and 20, where the agent quickly learns essential rules and patterns. This phase shows a steep learning curve, transitioning from rudimentary knowledge to a more strategic approach. From episodes 20 to 60, rewards fluctuate but generally trend upwards, indicating the agent’s ongoing adaptation and refinement of strategies. After episode 60, reward variability stabilizes, with values consistently around 3 to 4, suggesting developing a reliable policy. This stability indicates well-established learning, although minor adjustments continue as the agent optimizes its strategies to meet evolving challenges and opportunities for further enhancement.

#### 4.3.2. Tumor Detection

The proposed tumor detection model is compared with five models, DEHA-Net [64], DS-CMSF [65], DA-Net [66], R-CNN [67], PN-SAMPM [68], whose the results are shown Table 3. Within the competitive landscape, DEHA-Net sets a high standard with an IoU of 0.863, signifying a close match with the ground truth in tumor detection. DS-CMSF follows suit with an impressive IoU of 0.842. There is a notable drop in IoU for DA-Net, R-CNN, and PN-SAMPM, with scores of 0.790, 0.768, and 0.752, respectively. This descending trend might imply a diminishing accuracy in capturing the full extent of the tumors. Examining the precision of the tumor boundaries, DEHA-Net again takes the lead with the lowest HD of 0.726, which suggests that its predicted tumor edges are nearest to those in the actual medical images. The subsequent models, DS-CMSF, DA-Net, R-CNN, and PN-SAMPM, exhibit incrementally higher HD values, pointing to less precise boundary detections. The proposed model, however, outperforms all with an IoU of 0.901, surpassing DEHA-Net, the closest competitor, by 4.4%. This indicates that the proposed model detects the presence of tumors more accurately and provides a segmentation that significantly overlaps with the verified ground truth. Moreover, the proposed model achieves an HD of 0.625, an improvement of 13.9% over the best-performing existing model, DEHA-Net. This metric emphasizes the enhanced capability of the proposed model to delineate tumor boundaries with greater exactitude.

The statistical significance shows that the proposed model substantially outperforms existing models in tumor detection, with *p*-values < 0.05 indicating strong statistical significance. For instance, the proposed model’s IoU of 0.901 and HD of 0.625 compared favorably against the next best model, DEHA-Net, which recorded an IoU of 0.863 and HD of 0.726. The confidence intervals for these metrics further reinforce the superiority of the proposed model, illustrating robust improvements in both IoU and HD measures. These data substantiate the claim of our model’s superiority in precise tumor detection.

**Figure 5:**
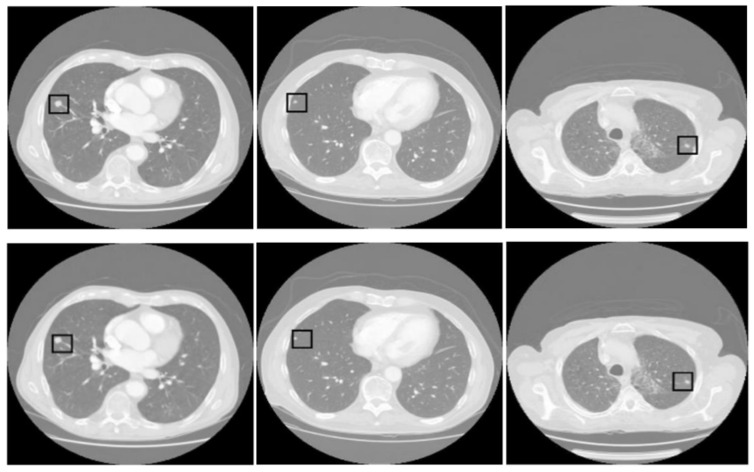
Comparative analysis of tumor detection. The top row shows the ground truth, while the bottom row presents the model predictions. The black square in every sample shows the tumor extracted by the proposed model.

Figure 5 presents three pairs of images generated by the proposed tumor detection model, where the ground truth annotations are displayed in the top row, and the outputs of the model are shown in the bottom row. A collective analysis of these images suggests that the proposed model demonstrates a commendable level of precision in pinpointing the location of lung tumors. Across the set, the regions marked by the model align well with the ground truth indicators, denoted by squares. This alignment indicates that the model has successfully learned to identify the critical features of lung tumors from CT scans and can consistently distinguish tumor tissue from surrounding anatomical features.

#### 4.3.3. Three-Dimensional Reconstruction

In our examination of various 3D reconstruction models, we have conducted an in-depth analysis of six distinctive methods: YOLOv4 [20], GAN-LSTM-3D [22], GAN-GK-LSTM [55], GAN-ResNet-3D [52], ANN-GGO [53], alongside our proposed model. To further assess our model, we conducted ablation studies to evaluate the impact of various components: Proposed w/o VGG-16, Proposed w/o SP, Proposed with GANDA, and Proposed with AEDA. The Proposed w/o VGG-16 configuration eliminates the VGG-16 layer, allowing images to be fed directly into the LSTM. The Proposed w/o SP variant does not utilize stratified partitioning for hyperparameter tuning. Additionally, the Proposed with GANDA and Proposed with AEDA models incorporate GAN and autoencoder (AE) for data augmentation purposes. The evaluation results are shown in Table 4.

Within the existing models, YOLOv4 takes the lead, showcasing the lowest values for HD at 1.146 and Euclidean Distance (ED) at 1.236, which serve as indicators of reconstruction accuracy. GAN-LSTM-3D trails just behind, with only marginally higher metrics. A more noticeable increase in ED is seen with GAN-GK-LSTM, particularly its rise to 1.494, signifying a decrease in volumetric precision. GAN-ResNet-3D and ANN-GGO, on the other hand, report the highest HD and ED values, with ANN-GGO deviating most substantially from the actual tumor shapes, as reflected by its HD of 2.630 and ED of 2.023. This diverse performance spectrum suggests that while some models approach the accuracy of our proposed model, there is room for improvement. The proposed model establishes a new standard, exhibiting HD and ED values of 0.986 and 1.126, respectively, thereby eclipsing the performance of all comparator models. Relative to YOLOv4, the nearest rival, our model demonstrates an improvement of approximately 13.9% in HD and 8.9% in ED. The advancements are even more striking against ANN-GGO—the model with the least accuracy among those compared—where we observe improvement rates of 62.5% in HD and 44.3% in ED. This pronounced differentiation emphasizes the refined capability of our model to delineate the complex structures of lung tumors with impressive precision.

The Proposed w/o VGG-16 model showed a significant decrease in performance, where omitting VGG-16 and directly inputting images into LSTM led to increased HD and ED values of 1.342 and 1.438, respectively. This decrease highlights VGG-16’s essential role in extracting image features, which affects the model’s accuracy in 3D lung tumor reconstruction. VGG-16 crucially enhances the interpretation of complex medical images, which could be more effectively handled by the LSTM alone. Additionally, eliminating stratified partitioning from hyperparameter tuning resulted in minor performance reductions, with HD and ED values of 0.975 and 1.186, respectively. This reflects a slight decrease in performance, indicating that while stratified partitioning supports robust training through balanced class representation, its absence subtly affects precision but does not critically reduce overall model effectiveness. Incorporating GAN for data augmentation (Proposed with GANDA) resulted in HD and ED values close to the base model, at 0.983 and 1.132, demonstrating that GANs effectively replicate real data variations and support accurate tumor reconstruction. Conversely, using an autoencoder for data augmentation (Proposed with AEDA) resulted in HD and ED values of 1.035 and 1.205, decreasing performance. Although AEDA enhances data representation, it may not fully capture the critical nuances needed for optimal 3D tumor reconstruction.

The statistical significance shows that the proposed model significantly outperforms the comparator models regarding HD and ED, with *p*-values consistently below 0.05 across all comparisons. These low *p*-values confirm that the differences in performance metrics between the proposed and existing models are not due to random chance, indicating a genuine improvement in 3D tumor reconstruction. The robust statistical analysis supports the claim of superiority of the proposed model over traditional approaches.

Table 5 highlights the computational efficiency of various 3D reconstruction models, a critical factor for applications requiring rapid processing. It provides a comparative analysis of runtime and GPU usage, offering key insights for those in the medical imaging and 3D reconstruction fields. The table shows YOLOv4 as moderately efficient with a runtime of 2235 s, indicating a balance between complexity and speed. In contrast, GAN-ResNet-3D has a much higher runtime of 3895 s, suggesting it may not be ideal for time-sensitive applications due to its complex processing. On the GPU usage front, GAN-LSTM-3D uses 10.2 GB, indicating efficiency, while GAN-GK-LSTM, the highest at 15.6 GB, suggests greater complexity. Our proposed model has a balanced runtime of 2475 s and uses 10.7 GB of GPU. It shows a good trade-off between performance and resource usage, making it adaptable for various clinical settings, even those with limited computational resources.

**Figure 6:**
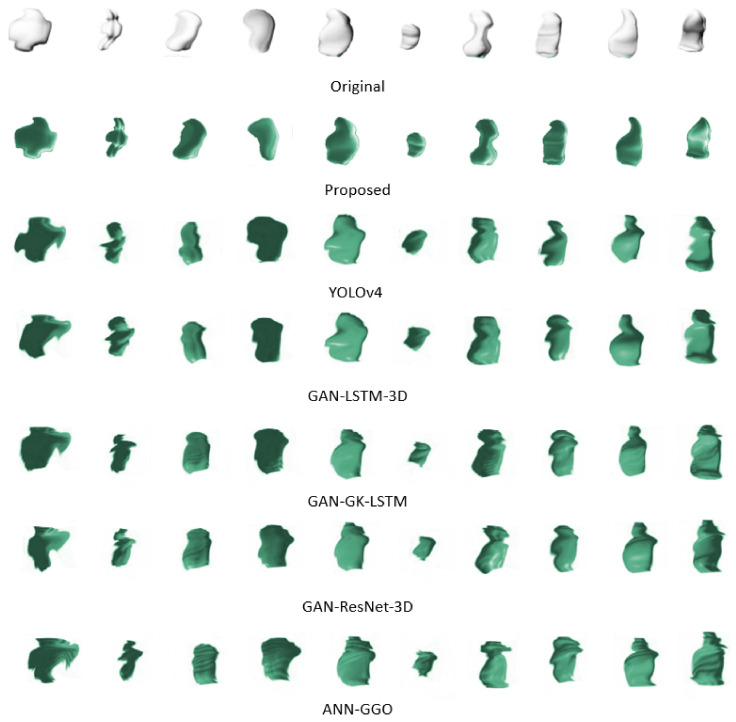
Comparison of original and reconstructed 3D tumor shapes highlighting the effectiveness of the proposed reconstruction method in preserving boundary smoothness.

Figure 6 presents a side-by-side comparison of the original 3D configurations of ten tumor samples against their digitally reconstructed counterparts. This juxtaposition underscores the complexities involved in replicating the seamless contours that characterize the true forms of the tumors. Traditional methods often falter at this juncture, unable to faithfully reproduce the smooth perimeters, which is crucial for precise reconstructions. However, our proposed method effectively navigates these common pitfalls. Employing advanced techniques and refined algorithms, it adeptly retains the smoothness of the tumor boundaries, thereby ensuring reconstructions that are both accurate and true to the original shapes. This achievement marks a substantial leap forward in 3D tumor imaging reconstruction.

Our investigation into the performance of diverse pre-trained models on 3D lung tumor reconstruction has revealed significant variations in accuracy. The experiment encompassed models such as AlexNet, GoogleNet, ResNet, DenseNet, and MobileNet, with VGG-16 as the reference model. According to Table 6, the precision of VGG-16 emerges as the highest, evidenced by its lowest scores in both HD and ED. The contrast is striking when we compare the improvement in precision that VGG-16 offers to that of other models. The HD and ED of AlexNet are elevated by approximately 72.5% and 29%, respectively, over those of VGG-16, underscoring the enhanced capability of the latter to detail tumor contours precisely. As we progress through models from GoogleNet to MobileNet, there is a noticeable regression in precision. The HD and ED measurements of these models exceed those of VGG-16 by substantial margins, ranging from 88% to over 100% for HD and 59% to 104% for ED. This trend makes it clear that the sophistication of the architecture of a model does not inherently translate to superior performance in specialized tasks such as 3D tumor reconstruction. Most strikingly, MobileNet, which is optimized for efficiency on devices with limited computational power, shows more than a doubling of the HD and ED values compared to those of VGG-16, translating to a decline in performance by over 100%. These results illustrate the compromise between efficiency and precision, with the streamlined structure of MobileNet sacrificing the latter.

**Figure 7:**
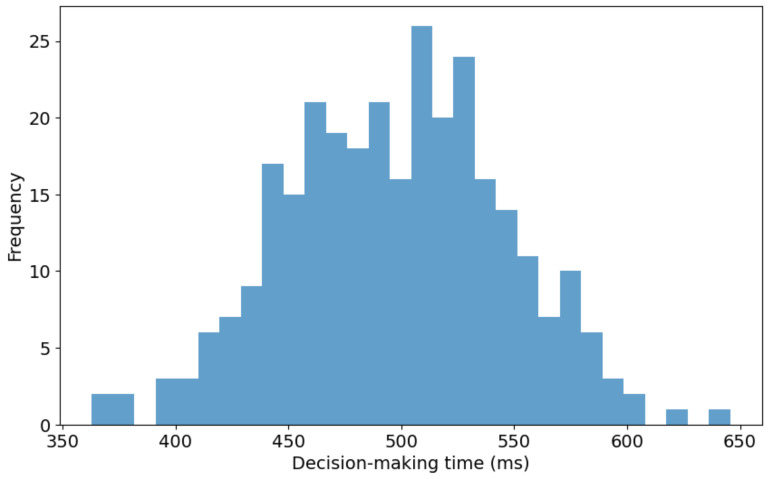
Distribution of decision-making times for 3D reconstruction in RTB environments.

Figure 7 presents the distribution of decision-making times for the proposed 3D reconstruction model utilized in real-time bidding (RTB) environments. The histogram illustrates decision-making times ranging from approximately 350 milliseconds (ms) to 650 ms. A noticeable concentration of decision times falls between 500 ms and 550 ms, signifying the modal interval where the model demonstrates optimal efficiency. The right-skewed distribution suggests that while most decision-making instances are grouped around the median range, fewer significantly prolonged decision times extend toward the upper limit of 650 ms. This skew indicates that the model occasionally experiences delays, possibly due to complex input data or computational constraints within the RTB system. Furthermore, the wide range of the distribution, covering a 300 ms interval, indicates a considerable variability in the model’s response times across different RTB scenarios. This variation could stem from inherent differences like the input data or the model’s computational process dynamics.

#### 4.3.4. Analysis of Generalization

To rigorously assess the generalization capabilities of our proposed model, we conducted experiments on the LUng Nodule Analysis (LUNA16) dataset [55] and the Medical Imaging and Data Resource Center (MIDRC) [69], comparing lung segmentation, tumor detection, and 3D reconstruction performance against other models. The results for these categories are detailed in Table 7, Table 8 and Table 9 for both the LUNA16 and MIDRC datasets. For lung segmentation, our model significantly outperformed competitors such as Deeplabv3plus, U-Net, and U-Net++ on both datasets, achieving an IoU 22% higher than Deeplabv3plus on the LUNA16 dataset and 20% higher on the MIDRC dataset. Its HD was reduced by 41% on LUNA16 and 39% on MIDRC, demonstrating superior precision in segmenting lung tissues, which is critical for early detection and accurate medical assessments. In tumor detection, our model excelled on both datasets, outperforming advanced models like DEHA-Net and DS-CMSF with higher IoU and significantly lower HD. It improved IoU by approximately 4.5% over DEHA-Net on LUNA16 and by 4.3% on MIDRC, and it reduced HD by 14% on LUNA16 and 12% on MIDRC, showcasing its precise ability to delineate tumor boundaries. In 3D tumor reconstruction, our model was significantly superior, outperforming GAN-based approaches like GAN-LSTM-3D and GAN-GK-LSTM on both datasets. It improved HD by 21% compared to GAN-LSTM-3D on LUNA16, indicating highly accurate reproduction of 3D tumor structures, and by 20% on MIDRC. These findings consistently underscore our model’s ability to maintain a high performance across different datasets, affirming its strong generalization capability and suitability for deployment in diverse clinical environments.

#### 4.3.5. Discussion

The proposed model in this article offers an innovative approach to lung cancer detection by leveraging GANs, LSTM networks, and RL algorithms to tackle significant challenges, including class imbalance. We meticulously executed experiments that underscored the superiority of our model compared to existing advanced technologies and conducted ablation studies to pinpoint the distinct contributions of each component. Table 10 provides a detailed comparative analysis between our method and other existing methods. Our findings reveal that the model excels in lung segmentation tasks, achieving high IoU and low HD, outperforming conventional models such as Deeplabv3plus, U-Net, and U-Net++. The integration of RL adapts the learning process dynamically, which is crucial for effectively managing the class imbalance prevalent in lung imaging datasets. This enhancement not only boosts the accuracy of lung segmentation but also extends the model’s utility across varied imaging scenarios. In tumor detection, the model’s innovative loss function within the GAN framework significantly refines its ability to delineate tumor boundaries, providing substantial improvements over high-performance models such as DEHA-Net and DS-CMSF. This capability is critical for detecting tumors with subtle and intricate imaging patterns. Furthermore, the advanced processing abilities of the LSTM networks significantly improve the model’s performance in the 3D reconstruction of lung tumors, facilitating the creation of detailed and accurate 3D tumor models crucial for effective treatment planning. This advancement represents a significant progression beyond traditional 3D techniques, including YOLOv4 and various GAN-based approaches. These results collectively validate our proposed model’s effectiveness and underscore its potential to transform existing lung cancer detection and treatment planning practices.

**Figure 8:**
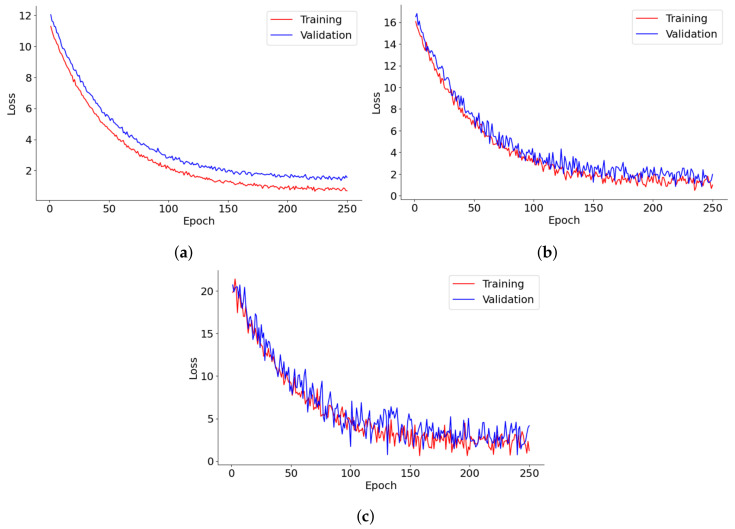
Loss trends in (**a**) lung segmentation, (**b**) tumor detection, and (**c**) 3D reconstruction models over 250 epochs.

Figure 8 provides a detailed depiction of the loss progression over 250 epochs for lung segmentation, tumor detection, and 3D reconstruction models. Each graph tracks the training and validation loss, shedding light on the dynamics of model learning and its stability over time. In lung segmentation, training, and validation, losses decrease swiftly during the early epochs before stabilizing, demonstrating effective learning with minimal risk of overfitting. The close alignment of training and validation losses throughout the process suggests that the model is well-calibrated and generalizes effectively to new data. For tumor detection, the loss initially plummets, followed by increased fluctuations in the validation loss as training progresses. This pattern may reflect the model’s sensitivity to specific tumor characteristics or variations in the validation data, underscoring potential areas for further optimization or the need for more diverse training examples. The 3D reconstruction model’s loss curve also trends downward steadily, albeit with noticeable volatility in the validation loss. This suggests difficulties in achieving convergence, potentially due to the complexities of processing 3D data or a lack of sufficient diversity in the training data. The frequent oscillations in the validation loss further imply challenges in handling anatomical variations that are not well-represented in the training dataset.

**Figure 9:**
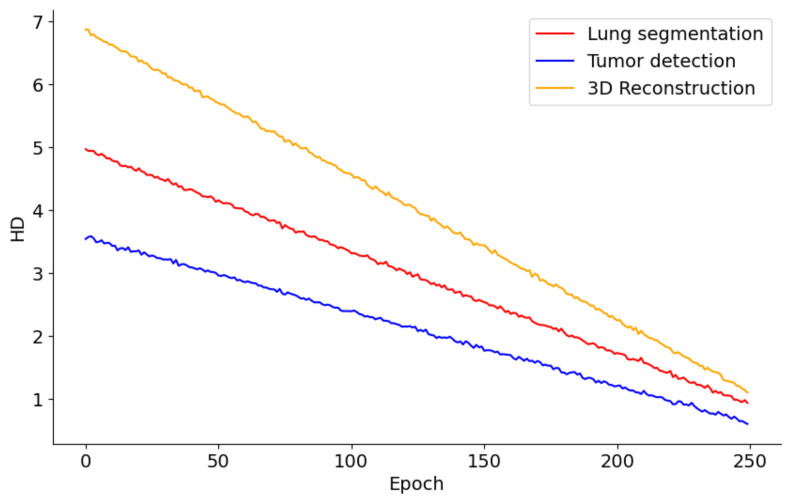
HD metric trends in lung segmentation, tumor detection, and 3D reconstruction over 250 epochs.

Figure 9 presents the performance trends across 250 epochs for models involved in lung segmentation, tumor detection, and 3D reconstruction utilizing the HD metric. The lung segmentation model shows a consistent reduction in HD throughout the 250 epochs, signaling a steady enhancement in the model’s precision in identifying lung boundaries relative to surrounding tissues. This improvement implies that the model is effectively learning from the training data and becoming increasingly adept at accurately distinguishing lung tissue over time. In contrast, the tumor detection curve demonstrates a sharper decline, especially in the initial epochs. This rapid improvement likely reflects the model’s sensitivity to changes in feature representation or adjustments to the learning rate, which are crucial for detecting smaller and irregularly shaped tumors. The pronounced steepness early in the training indicates that initial adjustments or learning of features significantly impact the model’s performance, with further refinement occurring in later epochs. Meanwhile, the 3D reconstruction process also exhibits a considerable reduction in HD, albeit slower than tumor detection. This slower rate of improvement may stem from the complexities involved in reconstructing three-dimensional structures from segmented images, necessitating more subtle learning and parameter adjustments. Nonetheless, the persistent downward trend indicates that the model is incrementally improving its ability to accurately reconstruct the 3D structures of lungs and tumors.

The inclusion of GANs in our model is strategic, leveraging their powerful ability to generate realistic images by pitting two neural networks against each other: a generator that creates images and a discriminator that evaluates them. This dynamic setup enables the generator to produce increasingly accurate representations of the desired output, in this case, lung tissues and tumors. GANs are particularly suited for medical image analysis due to their proficiency in handling complex image data and learning detailed and nuanced representations crucial for accurate medical diagnoses. The adversarial training ensures that the generated images are realistic and consistent with the diverse range of appearances lung tumors can exhibit in CT scans. By employing GANs, our model benefits from their robust image generation capabilities, ensuring that the segmentation and reconstruction of lung tumors are both precise and reliable, which is vital for the early detection and treatment planning of lung cancer.

RL is a critical component of our model, particularly in training the GAN used for lung segmentation. The inclusion of RL is driven by its ability to handle the complex, dynamic environments typical of medical imaging data, especially when dealing with imbalanced datasets such as lung CT images, where non-lung pixels vastly outnumber lung pixels. RL operates on agents learning to make decisions by performing actions in an environment to achieve cumulative rewards. In our GAN model, the generator acts as an agent that receives feedback from the discriminator (environment), which assesses the generated lung segmentation against the real data. The RL approach allows the generator to learn more nuanced strategies for producing realistic segmentations, beyond mere imitation of the training data, by rewarding the generator for segmenting lung regions that challenge the discriminator to distinguish from real lung tissues. The decision to use RL in training the GAN model is motivated by the need to address the class imbalance problem effectively. Traditional training methods might lead the model to overlook minority classes (lung pixels) due to their lesser presence in the training data. RL addresses this by assigning higher rewards for correctly identifying lung pixels, encouraging the model to pay more attention to these critical areas. This method enhances the sensitivity of the model to lung regions, improving segmentation accuracy and, consequently, the reliability of subsequent tumor detection and 3D reconstruction.

The theoretical implications of our research are significant, marking a substantial advancement in medical imaging and automated disease diagnosis. By integrating state-of-the-art machine learning techniques such as GANs, LSTM networks, and the VGG16 algorithm, our model sets a new benchmark in lung tumor detection and 3D reconstruction. Using GANs for segmentation and reconstruction introduces an innovative approach that leverages their generative capabilities to produce highly accurate depictions of lung tumors. Furthermore, applying LSTM networks to process sequential CT scan data underscores the potential of recurrent neural networks in preserving and utilizing temporal information, which is essential for constructing accurate 3D tumor models. Incorporating the VGG16 algorithm enhances the ability of the model to extract detailed and relevant features from complex medical images, laying a solid foundation for the accurate segmentation and detection of lung tumors. This research contributes to improving lung cancer diagnostic processes. It provides a versatile framework that could be adapted for other types of cancer and diseases, showcasing these advanced machine-learning techniques’ broad applicability and impact in healthcare.

The limitations of the proposed model are as follows:

- Data diversity and availability: One of the main limitations of our model is its dependence on the availability and diversity of training data. Medical imaging datasets, especially those for lung cancer, can vary widely in terms of quality, resolution, and the types of imaging equipment used. This variability can affect the ability of the model to generalize across clinical settings and patient populations. To mitigate this limitation, we recommend expanding the training dataset with images from various sources, including hospitals and imaging centers, to cover a broader spectrum of cases and conditions. Additionally, advanced data augmentation techniques can artificially enhance the dataset, introducing variations that simulate different imaging conditions and tumor characteristics, thereby improving the robustness and generalizability of the model.
- Computational complexity: The sophisticated architecture of our model, comprising multiple deep learning components such as GANs and LSTM networks, necessitates substantial computational resources for training and inference. This requirement could limit the accessibility and practicality of the model, especially in resource-constrained environments. To address this challenge, model optimization techniques such as network pruning, quantization, and knowledge distillation can be explored to reduce the model’s computational footprint without significantly compromising its performance. Additionally, adopting cloud computing and specialized hardware accelerators like GPUs and TPUs can offer scalable and cost-effective solutions for deploying the model in diverse clinical settings.
- Interpretability and explainability: The black-box nature of deep learning models, including ours, poses a challenge in clinical applications where understanding the rationale behind diagnostic predictions is crucial. Enhancing the interpretability of the model can foster trust among clinicians and patients by providing insights into the decision-making process. Techniques such as attention mechanisms, which highlight the regions of the image most influential in the predictions of the model, and model-agnostic explanation methods like LIME (Local Interpretable Model-agnostic Explanations) and SHAP (Shapley Additive explanations) can be integrated into our model. These methods can help elucidate the features and patterns the model relies on for segmentation and tumor detection, making its predictions more transparent and understandable for clinical practitioners.
- Adaptation to evolving clinical practices and imaging technologies: The rapid pace of advancement in medical imaging technologies and clinical practices presents a challenge to maintaining the relevance and effectiveness of our model. As new imaging modalities and techniques emerge, the model must be adaptable to leverage these advancements and incorporate new data types. Continuous learning and model updating strategies can be implemented to allow the model to evolve in response to further information and changing clinical environments. Transfer learning techniques can facilitate the integration of new data types and imaging modalities into the model, enabling it to stay current with the latest developments in medical imaging. By ensuring the adaptability of the model, we can maintain its efficacy and utility in clinical practice, supporting ongoing improvements in lung cancer diagnosis and treatment planning.

## 5. Conclusions

This paper presented an innovative approach for reconstructing 3D lung tumor images using a combination of GAN, LSTM, and VGG16 algorithms. The method addressed the challenge of accurately segmenting lung tissues in CT scans, which was complicated by many non-lung pixels, leading to potential classifier imbalance. This issue was mitigated using a GAN model trained with an RL-based algorithm, enhancing segmentation accuracy. Additionally, a second GAN model with a novel loss function was introduced to improve the precision of tumor detection within the segmented areas. Following successful detection, the VGG16 algorithm was used for feature extraction, which was then processed by an LSTM network for 3D reconstruction using a GAN equipped with dilated convolution layers. This setup allowed for the detailed capture of contextual information, ensuring an accurate tumor reconstruction. The effectiveness of the method was demonstrated through rigorous comparisons with established techniques on the LIDC-IDRI dataset, showcasing its potential to advance early lung cancer detection efforts significantly.

In future developments, we aim to improve our model’s adaptability and real-world performance by implementing real-time data feedback mechanisms. These mechanisms will dynamically adjust essential hyperparameters in response to current performance metrics and environmental changes, enhancing our reinforcement learning framework. Plans include integrating adaptive learning rates and automated hyperparameter tuning algorithms that respond to real-time data. Additionally, we aim to develop a simulation environment that mirrors real clinical settings, which will help us test and evaluate the impact of hyperparameter adjustments on the model’s generalizability and effectiveness. This will be vital for deploying our model in diverse clinical scenarios, ensuring it delivers reliable and effective results regardless of data variability. As another future work, we plan to enhance the interpretability of our model by integrating attention mechanisms that can highlight influential features for diagnostic predictions, aiding clinicians in understanding the decision-making process. Additionally, we will incorporate model-independent interpretation methods such as LIME and SHAP to improve transparency. This approach will enable clinicians and researchers to visually confirm and understand model predictions, ensuring the outputs are reliable and interpretable in real-world healthcare settings.

## Figures and Tables

**Table 1:** Hyperparameters for lung segmentation, tumor detection, and 3D reconstruction models.

Hyperparameter	Range	Optimal Value
Lung segmentation		
Learning rate (G)	0.0001–0.1	0.01
Number of layers (G)	5–20	12
Learning rate (D)	0.0001–0.1	0.01
Number of layers (D)	5–20	12
λ	0–1	0.4
Batch size	16–128	64
Momentum	0.8–0.99	0.9
Weight decay	0.00001–0.001	0.0001
Tumor detection		
Learning rate (Mask R-CNN)	0.00001–0.01	0.001
RPN anchor scales	32–512	128
RPN anchor ratios	0.5–2	1
ROI positive ratio	0.2–0.5	0.33
Bounding box refinement std	0.01–0.1	0.05
Mask threshold	0.5–0.7	0.6
3D reconstruction		
Learning rate (GAN)	0.00001–0.01	0.0002
Number of LSTM units	100–1000	512
LSTM layers	1–5	3
Dropout rate (LSTM)	0.0–0.5	0.25
Beta1 (Adam optimizer)	0.5–0.9	0.5
Beta2 (Adam optimizer)	0.999	0.999
Batch size	8–64	16

**Table 2:** Performance metrics of the proposed algorithm and various deep learning algorithms for lung segmentation on the LIDC-IDRI dataset.

Method	IoU	HD
Deeplabv3plus [30]	0.798	1.638
U-Net [33]	0.762	1.914
U-Net++ [32]	0.750	1.748
LDDNet [61]	0.662	0.957
LGAN [62]	0.632	0.940
3D Res U-Net [63]	0.623	2.835
Proposed w/o RL	0.726	1.205
Proposed	0.972	0.925

**Table 3:** Performance metrics of various deep learning algorithms for tumor detection on the LIDC-IDRI dataset.

Method	IoU	HD
DEHA-Net [64]	0.863	0.726
DS-CMSF [65]	0.842	0.769
DA-Net [66]	0.790	0.871
R-CNN [67]	0.768	1.206
PN-SAMPM [68]	0.752	1.426
Proposed	0.901	0.625

**Table 4:** Performance metrics of the proposed model versus comparator models for lung tumor 3D reconstruction on the LIDC-IDRI dataset.

Method	HD	ED
YOLOv4 [20]	1.146	1.236
GAN-LSTM-3D [22]	1.163	1.270
GAN-GK-LSTM [55]	1.182	1.494
GAN-ResNet-3D [52]	2.260	1.852
ANN-GGO [53]	2.630	2.023
Proposed w/o VGG-16	1.342	1.438
Proposed w/o SP	0.975	1.186
Proposed with GANDA	0.983	1.132
Proposed with AEDA	1.035	1.205
Proposed	0.986	1.126

**Table 5:** Computational efficiency comparison of models.

Method	Runtime (s)	GPU Usage (GB)
YOLOv4 [20]	2235	12.5
GAN-LSTM-3D [22]	2560	10.2
GAN-GK-LSTM [55]	2185	15.6
GAN-ResNet-3D [52]	3895	13.9
ANN-GGO [53]	2069	13.7
Proposed	2475	10.7

**Table 6:** Performance of different pre-trained models for the reconstruction of 3D lung tumors.

Model	HD	ED
AlexNet	2.462	1.832
GoogleNet	2.682	2.259
ResNet	2.756	2.349
DenseNet	2.852	2.536
MobileNet	2.920	2.896

**Table 7:** Performance metrics of various deep learning algorithms for lung segmentation on the LUNA16 and MIDRC datasets.

Method	LUNA16		MIDRC	
	IoU	HD	IoU	HD
Deeplabv3plus [30]	0.780	1.750	0.742	1.953
U-Net [33]	0.740	1.960	0.702	2.124
U-Net++ [32]	0.730	1.850	0.692	2.063
LDDNet [61]	0.650	1.000	0.611	1.232
LGAN [62]	0.620	0.970	0.582	1.126
3D Res U-Net [63]	0.610	2.950	0.574	3.124
Proposed	0.955	1.035	0.926	1.146

**Table 8:** Performance metrics of various deep learning algorithms for tumor detection on the LUNA16 and MIDRC datasets.

Method	LUNA16		MIDRC	
	IoU	HD	IoU	HD
D DEHA-Net [64]	0.850	0.760	0.823	0.782
DS-CMSF [65]	0.830	0.790	0.801	0.810
DA-Net [66]	0.780	0.910	0.753	0.942
R-CNN [67]	0.760	1.250	0.736	1.2713
PN-SAMPM [68]	0.740	1.450	0.713	1.4796
Proposed	0.880	0.650	0.868	0.672

**Table 9:** Performance metrics of the proposed model versus comparator models for lung tumor 3D reconstruction on the LUNA16 and MIDRC datasets.

Method	LUNA16		MIDRC	
	HD	ED	HD	ED
YOLOv4 [20]	1.190	1.300	1.396	1.512
GAN-LSTM-3D [22]	1.200	1.350	1.458	1.523
GAN-GK-LSTM [55]	1.250	1.600	1.512	1.963
GAN-ResNet-3D [52]	2.300	1.900	2.863	2.141
ANN-GGO [53]	2.700	2.100	2.863	2.341
Proposed	0.942	1.025	0.982	1.179

**Table 10:** Comparative analysis of the proposed method against existing models.

Model	Comparison	Improvement (%)	Reason for Superiority
Lung segmentation	The proposed method obtained IoU = 0.972, HD = 0.925, significantly outperforms existing models such as Deeplabv3plus [30], U-Net [33], and U-Net++ [32], LDDNet [61] in terms of IoU and HD metrics, demonstrating a notable increase in segmentation accuracy and a decrease in Hausdorff distance, which indicates more precise contour adherence.	21.9% (IoU), 43.6% (HD)	Using RL enhances the model’s capability to effectively manage the classifier imbalance, improving accuracy in segmenting lung tissues.
Tumor detection	Compared to other advanced models like DEHA-Net [64], DS-CMSF [65], our method achieves superior IoU and lower HD scores (IoU = 0.901, HD = 0.625), highlighting its enhanced capability to detect tumor boundaries.	4.4% (IoU), 13.1% (HD)	Enhanced detection accuracy results from using the novel loss function within the GAN model, which optimizes the delineation of tumor boundaries, surpassing traditional deep learning methods.
3D reconstruction	Our model excels in 3D reconstruction of lung tumors, showing the lowest HD and ED (HD = 0.986, ED = 1.126) compared to methods like YOLOv4 [20] and various GAN-based approaches. This illustrates the proposed model’s precision in reconstructing more accurate and detailed 3D tumor geometries.	13.9% (HD), 8.9% (ED)	The accuracy of 3D reconstructions is significantly enhanced due to the efficient underlying models for lung segmentation and tumor detection, combined with the sophisticated temporal processing capabilities of LSTM networks.

## Data Availability

Data will be made available on request.
